# POU2F1 Promotes Cell Viability and Tumor Growth in Gastric Cancer through Transcriptional Activation of lncRNA TTC3-AS1

**DOI:** 10.1155/2021/5570088

**Published:** 2021-06-28

**Authors:** Jixu Wang, Ke Xiao, Futao Hou, Lusheng Tang, Dan Luo, Gu Liu, Zhiqiang Wang

**Affiliations:** ^1^Key Laboratory of Medical Imaging and Artificial Intelligence of Hunan Province, Xiangnan University, Chenzhou 423000, China; ^2^Department of Gastroduodenal and Pancreatic Surgery, Hunan Cancer Hospital and the Affiliated Cancer Hospital of Xiangya School of Medicine, Central South University, Changsha, Hunan 410013, China; ^3^Department of General Surgery, Hunan Provincial People's Hospital, 61 West Jiefang Road, Furong District, Changsha, 410003 Hunan Province, China; ^4^Department of Vascular Surgery, Chenzhou First People's Hospital and the First Affiliated Hospital of Xiangnan University, 102 Luojiajing, Chenzhou, 423000 Hunan Province, China; ^5^Department of Gastrointestinal Surgery, Chenzhou First People's Hospital and the First Affiliated Hospital of Xiangnan University, 102 Luojiajing, Chenzhou, 423000 Hunan Province, China

## Abstract

POU domain, class 2, transcription factor 1 (POU2F1) is involved in the development of gastric cancer (GC). However, the molecular mechanism has not been fully elucidated. Here, we identified a novel lncRNA named TTC3-AS1 that was potentially regulated by POU2F1 and investigated their roles in GC progression. Bioinformatics analysis suggested that high expression of POU2F1 predicted poor prognosis in patients with GC. We further screened out an lncRNA TTC3-AS1 that may be transcriptionally activated by POU2F1 according to the JASPAR database, and POU2F1 and TTC3-AS1 were highly expressed in GC cells and tissues compared with normal controls (NCs). Function analysis revealed that both POU2F1 and TTC3-AS1 played oncogenic roles by promoting cell viability, migration, and invasion in GC. qRT-PCR analysis showed that POU2F1 improved the expression of TTC3-AS1 in GC cells, while TTC3-AS1 knockdown or overexpression had no effect on POU2F1 expression. The results of chromatin immunoprecipitation and DNA-affinity precipitation assays indicated that POU2F1 directly bound to the promoter region of TTC3-AS1 and activated its transcription. TTC3-AS1 knockdown neutralized the protumor effects of POU2F1 overexpression in GC cell lines as well as mouse models of GC, which suggested that TTC3-AS1 mediates the oncogenic function of POU2F1. In summary, POU2F1 promoted GC progression by transcriptionally activating TTC3-AS1; thus, this study provided a new perspective for the mechanism of GC progression.

## 1. Introduction

Due to the lack of early symptoms and rapid disease progression, gastric cancer (GC) has become one of the most frequent malignancies and the third leading cause of cancer deaths worldwide [1, 2]. Although most patients with early GC can be cured by surgery, more than half of patients with advanced GC suffer from cancer recurrence, even after curative gastrectomy [3]. The five-year overall survival rate of GC patients is approximately 40% [4, 5]. Characteristic progressive tumorigenesis and distant metastasis are the most important risk factors for poor prognosis of GC patients; hence, the five-year survival rate of GC patients with distant metastasis is only 5% [5]. Therefore, identifying the molecular mechanisms of tumorigenesis and distant metastasis in GC is urgently needed.

POU domain, class 2, transcription factor 1 (POU2F1), also known as octamer-binding transcription factor 1 (OCT-1), is a ubiquitous transcription factor involved in regulating the physiological and pathological processes of cancer cell, including cell cycle and differentiation, DNA damage repair, and glucose metabolism [6–9]. In addition, POU2F1 is implicated in immunity and inflammation, activity maintenance of tumor stem cells as well as tumorigenesis, and development through modulation of the expression of the tissue-specific target gene [10–14]. It has been reported that POU2F1 is highly expressed in various types of tumors, including osteosarcoma, gastric cancer, and head and neck squamous cell carcinoma [15–17]. POU2F1 is also an independent prognostic factor in gastric cancer. Moreover, knockdown of POU2F1 leads to apparent inhibition of tumor proliferation and invasion in head and neck squamous cell carcinoma, gastric cancer, and so on [17, 18]. Accumulated evidence has indicated that POU2F1 functions as an oncogene in tumor progression. However, the action mechanism of POU2F1 remains elusive.

lncRNAs are defined as noncoding RNAs >200 bp in length, with limited or no protein coding ability [19]. In the past, lncRNAs were considered as nonsense sequences in the transcription process, with no biological function. However, recent increasing studies have found that lncRNAs play crucial roles in multiple biological processes, including cell proliferation, apoptosis, and differentiation, as well as stem cell pluripotency [20, 21]. Furthermore, more lncRNAs have been identified as tumor oncogenes or suppressor genes through regulating various tumor-associated processes, including epithelial-mesenchymal transition and tumor metastasis and growth [21–25]. Therefore, identifying novel functional lncRNAs implicated in GC progression and elucidating their action mechanisms are important for the cognition and treatment of GC.

In this study, we demonstrated that POU2F1/TTC3-AS1 were highly expressed and predicted poor prognosis in GC patients. POU2F1 could directly bind to the promoter region of TTC3-AS1 and promote its expression. Function analysis revealed that POU2F1 promoted GC cell viability, migration and invasion, and tumor growth through upregulation of TTC3-AS1. Our study extended the knowledge of the POU2F1 mechanism in tumors and provided potential therapeutic targets for GC patients.

## 2. Materials and Methods

### 2.1. Bioinformatics Analysis

HCMDB (Human Cancer Metastasis Database, http://hcmdb.i-sanger.com/index) is an online database that provides comprehensive gene expression profiles of metastatic and primary tumor samples. Differentially expressed genes in metastatic tumor samples (*n* = 21) and primary tumor samples (*n* = 351) from HCMD-EXP00440 were evaluated by network analysis of the stomach adenocarcinoma samples in The Cancer Genome Atlas (TCGA-STAD). Then GEPIA (Gene Expression Profiling Interactive Analysis) platform was used to perform gene intersection analysis and prognostic analysis of the TCGA-EXP00440 database. Significantly different expression of mRNA in metastatic tumor samples was determined in accordance with *P* < 0.05, and cluster analysis of differentially expressed mRNAs was performed using the DAVID (Database for Annotation, Visualization, and Integrated Discovery).

### 2.2. Human Tissue Specimens

Tumor tissues and adjacent normal tissues were collected from 10 patients diagnosed with GC through pathological examination as well as surgery from July 2017 to July 2019. The study was approved by the independent Ethics Committee of Hunan Cancer Hospital and The Affiliated Cancer Hospital of Xiangya School of Medicine, and written informed consent was obtained from each patient. Patients received no treatment prior to the surgery. The tumor tissues were rapidly transferred into liquid nitrogen immediately after the surgery.

### 2.3. Cell Lines

The human gastric adenocarcinoma cell lines MGC803, BGC823, MKN28, MKN45, and SGC7901 as well as the normal gastric epithelial cell line (GES-1) were obtained from the Chinese Academy of Sciences Committee on Type Culture Collection Cell Bank (Shanghai, China). MGC803, BGC823, and MKN28 cells were cultured in Roswell Park Memorial Institute (RPMI) 1640 medium; GES-1 and SGC7901 cells were cultured in a Dulbecco's modified Eagle's medium (DMEM; GIBCO-BRL) supplemented with 10% fetal bovine serum (FBS), 100 U/ml penicillin, and 100 mg/ml streptomycin (Invitrogen, Carlsbad, CA, USA) at 37°C in 5% CO_2_.

### 2.4. Quantitative Real-Time Polymerase Chain Reaction (qRT-PCR)

Total RNA from tissues or cells was isolated using TRIzol reagent (Invitrogen) in accordance with standard protocols. RNA of 1 *μ*g was reverse-transcribed into cDNA using a reverse transcription kit (Takara, Otsu, Japan). qRT-PCR was performed using the ABI 7500HT Fast Real-Time PCR System (Applied Biosystems, Waltham, MA, USA) under the following reaction conditions: 95°C for 10 min, 40 cycles at 95°C for 15 s, 60°C for 30 s, and 72°C for 20 s. GAPDH was used as an internal control. Relative mRNA expression was quantified by the comparative 2^−△△CT^ method [26]. Primer sequences are shown in Table 1.

### 2.5. Cell Transfection

Cell transfection was performed using Lipofectamine 2000 (Invitrogen, Carlsbad, CA, USA) according to the provider's instructions. The POU2F1 siRNA and overexpression plasmids as well as TTC3-AS1 siRNA and overexpression plasmids were obtained from Ribobio (Guangzhou, China). The cells were transfected with a final concentration of 50 nM and then collected for further assays, and the incubation duration was 48 h. In xenograft growth assay, lentivirus-mediated POU2F1 or TTC3-AS1 siRNA was designed and transfected into the GC cell lines.

### 2.6. CCK-8 Assay

The cell viability of SGC7901 cells was detected using CCK-8 kits (Dojindo, Japan) in accordance with the standard protocol. SGC7901 cells were transfected and then seeded into a 96-well plate with a density of 3,000 cells per well. The cells were cultured at 37°C in a humid atmosphere with 5% CO_2_. After a certain period, cells in each well were incubated with 10 *μ*l CCK-8 solution for 2 h. Finally, the absorbance of cell solution in 96-well plates was detected using a microplate reader at 570 nm (VarioSkan, Thermo Fisher Scientific, MA, USA). All experiments were performed in triplicate, and the results were expressed as means ± SD.

### 2.7. Cell Invasion and Migration Assays

For cell invasion assay, transwell membranes (BD, Franklin Lakes, NJ, USA) were precoated with Matrigel. After transfection for 24 h, SGC7901 cells were resuspended in serum-free RPMI 1640 medium with a density of 1 × 10^6^/ml. The cell solution of 100 *μ*l was added into the upper transwell chamber, and the lower chamber was added with 500 *μ*l of 20% FBS-containing RPMI 1640 medium. After 48 h of incubation, the cells on the upper surface of transwell membranes were removed, and invasive cells on the lower surface were stained with crystal violet for 10 min. Positive stained cells were counted under a microscope (Olympus) at five random fields.

For wound healing assay, transfected cells were cultured in complete RPMI 1640 medium to 90% confluence. After that, the cell monolayers were scratched with a 10 *μ*l pipette tip to create a single-line wound. The cells were washed by PBS to remove suspended cells. Next, the cells were cultured in the serum-free medium at 37°C with 5% CO_2_ for 48 h. The wound area at 0 h and 48 h was observed using a microscopy and quantified using ImageJ software.

### 2.8. Western Blot

Total proteins from cells or tissues were extracted using a total protein extraction kit (NBP2-37853, Novus Biologicals, Littleton, CO, USA). The protein concentration of each sample was detected by a BCA (bicinchoninic acid) method. After denaturing, proteins were separated using 10% SDS-PAGE (sodium dodecyl sulfate-polyacrylamide gel electrophoresis) with 20 *μ*g per lane. Protein bands were then transferred to a PVDF (polyvinylidene fluoride) membrane and blocked in 5% fat-free milk for 4 h at room temperature. Sequentially, protein bands were incubated with primary antibodies at 4°C overnight and horseradish peroxidase- (HRP-) conjugated secondary antibodies for 1 h at room temperature. The primary antibodies were as follows: POU2F1 (1 : 500; catalog: 10387-1-AP; Proteintech Group, Inc., Wuhan, China), E-cadherin (1 : 1000; catalog: 20874-1-AP; Proteintech Group, Inc., Wuhan, China), and FN1 (1 : 800; catalog: 30506; ProMab Biotechnology, Shanghai, China). Goat anti-rabbit IgG or goat anti-mouse IgG was used as the secondary antibody (1 : 4000; Proteintech Group, Inc., Wuhan, China). *β*-Actin (1 : 2000; Catalog: 66009-1-Ig; Proteintech Group, Inc., Wuhan, China) was used as an internal control. ECL (Sigma-Aldrich, USA) was utilized to develop signals. Relative expression levels of proteins were normalized using ImageJ v1.46 software (National Institutes of Health).

### 2.9. DNA Affinity Precipitation Assay (DAPA)

5′-Biotinylated double-stranded oligonucleotides corresponding to wild-type or mutant sequences for predicting POU2F1 binding sites on TTC3-AS1 were designed and produced by Genescript (Nanjing, China). DAPA was performed as previously described [27] with some modifications. Briefly, the assays were performed in a final volume of 400 ml of buffer D (20 mM HEPES, 10% glycerol, 50 mM KCl, 0.2 mM EDTA, 1.5 mM MgCl_2_, 10 mM ZnCl_2_, 1 mM DTT, and 0.25% Triton X-100; pH 7.9), by mixing 4 mg of biotinylated double-stranded 31 WT oligonucleotides with 20 ± 30 mg of nuclear extracts. The mix was incubated on ice for 45 min and then was added to the buffer D with equilibrated streptavidin-coated magnetosphere particles (SMPs) (Promega). The mixture was incubated at room temperature for 2 h with continuous agitation. SMPs were then captured using the magnetic stand, and the supernatant was removed without disturbing the SMPs pellet. Particles were washed four times with buffer D, and the final pellet obtained was resuspended in 2 × SDS ± PAGE loading buffer and boiled for 5 min to uncouple the oligonucleotide bound proteins. After capturing the SMPs using the magnetic stand, the supernatant was loaded on SDS ± PAGE gel, and western blot analysis was performed.

### 2.10. Chromatin Immunoprecipitation Assay (ChIP)

The direct interaction between POU2F1 and the promoter region of TTC3-AS1 was demonstrated using a CHIP assay. The assay was performed using a Magna ChIP kit (Millipore, Bedford, MA, USA). In brief, SGC7901 cells were cross-linked by incubation with formaldehyde, and glycine was used to terminate the reaction. Nuclear DNA is fragmented into 200–500 bp through sonication. The chromatin extract was incubated with an antibody against POU2F1 or IgG (Millipore). After immunoprecipitation, the DNA-protein-antibody complex was separated, and the protein was removed. The purified DNA was analyzed using qRT-PCR. The IgG group and the input group were used as the negative control group and the positive control group, respectively.

### 2.11. Xenograft Growth Assay

Twelve BALB/c nude mice (5–6 weeks old, 20–25 g) were obtained from the Department of Laboratory Animals of Xiangya Hospital Central South University (Changsha, China). The mice were housed in a pathogen-free environment under standard feeding conditions. All procedures were approved by the independent Ethics Committee of Xiangya Hospital Central South University. The mice were anesthetized, and 2 × 10^6^ SGC7901 cells were transfected with a lentiviral vector containing NC sequence; POU2F1-OE or POU2F1-OE + TTC3-AS1-KD were subcutaneously injected into the back flank of mice. After xenograft for 10 days, tumor volume was measured every 3 days. At day 25, all the mice were sacrificed, and solid tumors were resected to measure weight or further analysis.

### 2.12. Immunohistochemistry (IHC) Analysis

The tumor tissues were embedded in paraffin and cut into 4 *μ*m slides. The antibodies against FN1 and Ki67 were obtained from Cell Signaling Technology. IHC analysis was performed as described in the previous study [28].

### 2.13. Dual-Luciferase Reporter Assay

**Figure 1:**
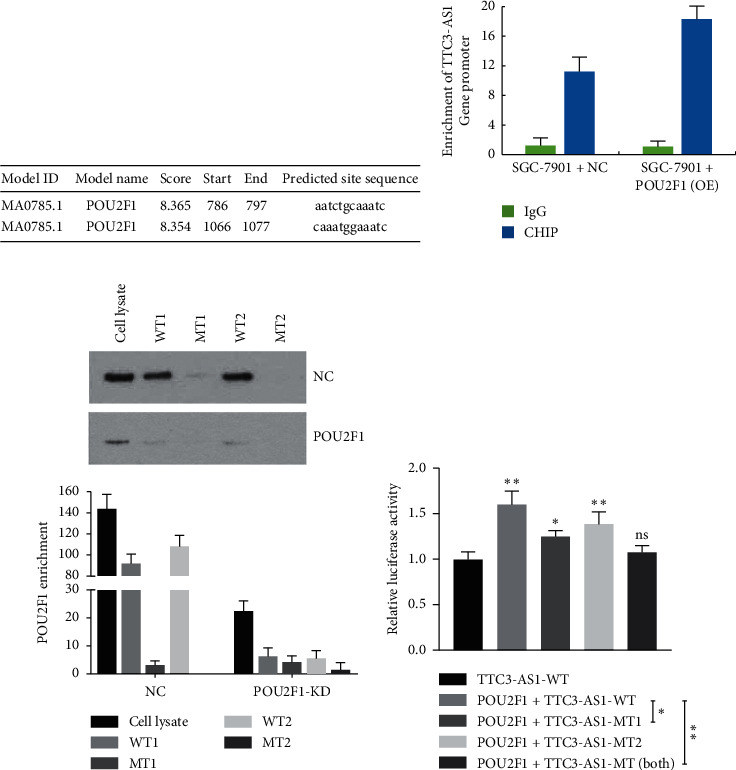
POU2F1 directly binds to the promoter region of TTC3-AS1. (a) The predicted POU2F1 binding sequences on the upstream of TTC3-AS1 promoter region. (b) ChIP and (c) DAPA assays were performed to reveal the affinity of POU2F1 to the TTC3-AS1 promoter in SGC7901 cells. (d) Luciferase reporter assay: luciferase reporter vectors TTC3-AS1-wild type (WT) and TTC3-AS1-mutant type (MT) including TTC3-AS1-MT1 (786–797 bp) and TTC3-AS1-MT2 (1,066–1,077 bp) were constructed. These luciferase reporter vectors were transfected into SGC901 cells together with the POU2F1 overexpression vector. All experiments were performed in triplicate. ^*∗*^*P* < 0.05 and ^*∗∗*^*P* < 0.01.

Luciferase reporter vectors, TTC3-AS1-wild type (WT) and TTC3-AS1-mutant type (MT), were constructed using PmirGLO (Promega, Madison, WI, USA). We predicted the potential POU2F1 binding sites in the TTC3-AS1 promoter region through the JASPAR database (https://jaspar.genereg.net), and two high-score POU2F1 binding sites positioned at 786–797 bp and 1,066–1,077 bp were identified (Figure 1(a)). Three TTC3-AS1-MT vectors constructed were based on the two binding sites POU2F1 potentially targeted in the TTC3-AS1 promoter region. In addition, the two POU2F1 binding sites were mutated simultaneously to construct the other luciferase reporter vector. The three mutant POU2F1 binding sites were generated using a Mut Express II Fast Mutagenesis Kit (Vazyme, Nanjing, Jiangsu, China). SGC901 cells were seeded into 24-well plates, and cotransfection of TTC3-AS1-WT or TTC3-AS1-MT vectors with POU2F1 overexpression plasmids into the cells was performed using Lipofectamine™ 2000 (Invitrogen, Carlsbad, California). The luciferase activity was detected 72 h after cotransfection using the Dual-Luciferase® Reporter Assay System (Promega, DLR™, E1960).

### 2.14. Statistical Analysis

All statistical analyses were performed using the IBM SPSS software (version 26.0; IBM SPSS Inc., Chicago, IL, USA). Survival curves were generated using the Kaplan–Meier method and assessed using the logrank test. Differences between the two groups were analyzed by either two-tailed Student's *t*-test or one-way ANOVA followed by post hoc Dunnett's test. *P* < 0.05 was considered to be statistically significant.

## 3. Results

### 3.1. Bioinformatics Analysis

**Figure 2:**
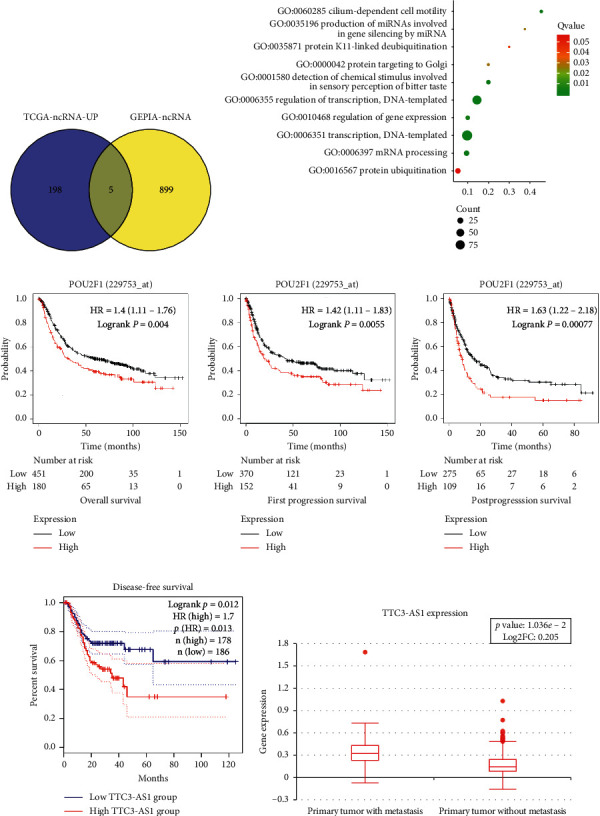
Analysis of differentially expressed genes in metastatic GC tumor samples and primary GC tumor samples. (a) 224 differentially expressed ncRNAs between metastatic tumor samples (*n* = 21) and primary tumor samples (*n* = 351) from the HCMD-EXP00440 database; 904 differentially expressed ncRNAs between GC tumor tissues and adjacent normal tissues correlated with GC prognosis; 5 lncRNAs (CATIP-AS2, TTC3-AS1, LINC01993, LINC01564, and LINC02015) upregulated in various cancers involving GC. (b) Cluster analysis of differentially expressed mRNAs using the DAVID database. (c) The correlation between POU2F1 expression and prognosis of GC patients from TCGA database. (d) The correlation between TTC3-AS1 expression and DFS of GC patients from the GEPIA platform. (e) TTC3-AS1 expression in primary tumors with metastasis and primary tumors without metastasis.

We first determined the differentially expressed genes between metastatic tumor samples (*n* = 21) and primary tumor samples (*n* = 351) from HCMD-EXP00440 by TCGA-STAD net analysis. In total, 18953 mRNAs, 3210 lncRNAs, and 2588 miRNAs were analyzed. The results showed that, compared with primary tumor samples, there were 224 differentially expressed ncRNAs (lncRNAs and miRNAs) in metastatic tumor samples, of which 203 upregulated and 21 downregulated. We further used the GEPIA platform to perform gene intersection analysis and prognostic analysis of the TCGA-EXP00440 database. As shown in Figure 2(a), TCGA-ncRNA-UP referred to above TCGA-STAD-derived 203 upregulated genes in metastatic tumor samples, compared with primary tumor samples. GEPIA-ncRNA referred to a summary of ncRNAs differentially expressed between GC tumor tissues and adjacent normal tissues as well as associated with prognosis of GC patients in the GEPIA database. It was found that 5 lncRNAs (CATIP-AS2, TTC3-AS1, LINC01993, LINC01564, and LINC02015) were upregulated in various types of cancers including GC (Figure 2(a)).

**Figure 3:**
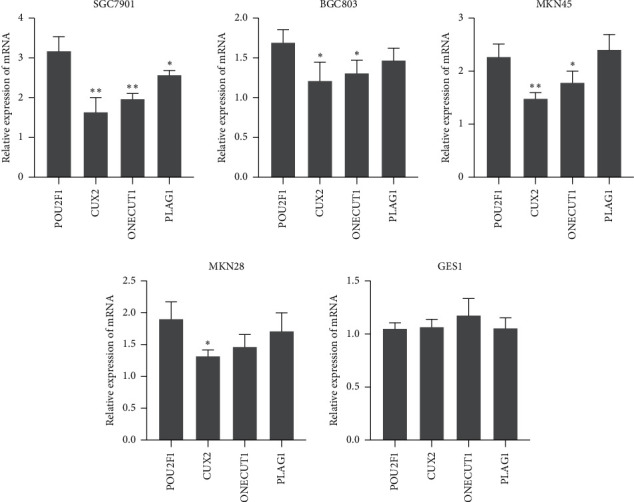
The expressions of 4 TFs (CUX2, POU2F1, PLAG1, and ONECUT1) were detected in 4 GC cell lines (SGC7901, MGC803, BGC823, and MKN28 ) and a normal gastric epithelial cell line, GES-1. ^*∗*^*P* < 0.05 and ^*∗∗*^*P* < 0.01 versus POU2F1 expression.

mRNA analysis indicated that 603 mRNAs were significantly upregulated in metastatic tumor samples, compared with primary tumor samples (*P* < 0.05, Figure 2(b)). Cluster analysis was performed to identify differentially expressed mRNAs through the DAVID database. The results revealed that transcription factors (TFs) correlated with signaling pathways were prominent, including 11 specific TFs (POU5F1, CUX2, TBX19, POU2F1, PLAG1, ZFP57, LMX1A, MZF1, BACH1, ONECUT1, and IRF9), implying that these TFs were involved in GC tumor metastasis. Among 11 specific TFs (POU5F1, CUX2, TBX19, POU2F1, PLAG1, ZFP57, LMX1A, MZF1, BACH1, ONECUT1, and IRF9), 4 TFs (CUX2, POU2F1, PLAG1, and ONECUT1) were identified to be able to predict poor prognosis in GC. Next, the expression of the 4 TFs was detected in 5 GC cell lines (MGC803, BGC823, MKN28, SGC7901, and normal GES-1). As shown in Figure 3, in general, POU2F1 expression was prominently higher compared with the expression of the other three TFs in GC cell lines. Next, we analyzed whether the 11 TF genes had potential binding regions on promoters of the above-mentioned 5 lncRNAs using the JASPAR database. We found that POU2F1 is potentially bound to the promoter region of TTC3-AS1 and LINC01564 through the JASPAR database. Meanwhile, no binding sites that POU2F1 potentially targeted were found in the promoter regions of CATIP-AS2, LINC01993, and LINC02015. Furthermore, TTC3-AS1 has higher prediction scores compared with LINC01564; thus, we selected TTC3-AS1 for further investigation.

The prognosis value of POU2F1 and lncRNA TTC3-AS1 in GC patients was evaluated by bioinformatic analysis. Multivariate analyses for overall survival (OV), disease-free survival (DFS), and progression-free survival (PFS) suggested that high expression of POU2F1 was associated with decreased OS, DFS, and PFS of GC patients in TCGA-STAD cohort (*P*=0.004, *P*=0.0055, and *P*=0.00077, respectively; Figure 2(c)). Moreover, GC patients with high expression of lncRNA TTC3-AS1 had a poor prognosis in terms of DFS (Figure 2(d)). There is a significant difference in TTC3-AS1 expression between metastatic and primary tumor samples (Figure 2(e)).

**Figure 4:**
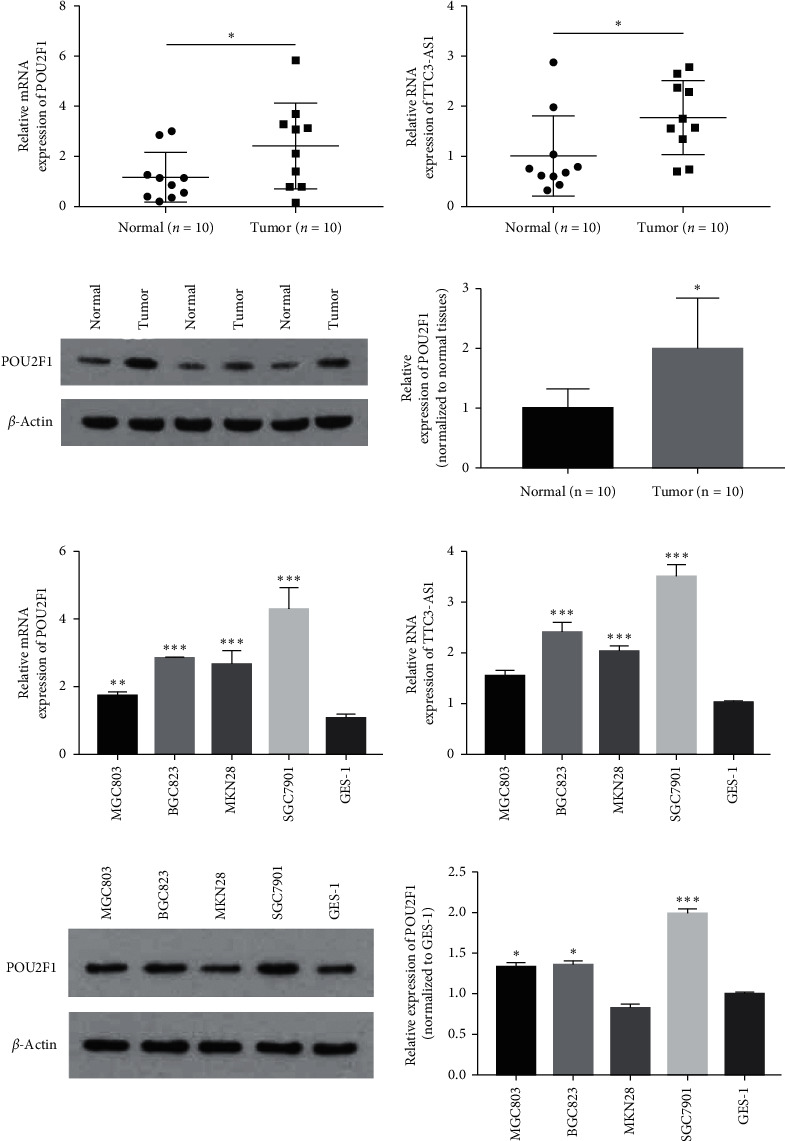
POU2F1 and TTC3-AS1 were upregulated in GC tumor tissues and cell lines. The RNA expression of (a) POU2F1 and (b) lncRNA TTC3-AS1 in GC tumor tissues and normal control tissues was detected using qRT-PCR. (c) POU2F1 protein expression in GC tumor tissues and normal control tissues was detected using western blot. The RNA expression of (d) POU2F1 and (e) lncRNA TTC3-AS1 in GC cell lines of MGC803, BGC823, MKN28, and SGC7901 and the normal gastric epithelial cell line (GES-1) was detected using qRT-PCR. (f) POU2F1 protein expression in GC cell lines of MGC803, BGC823, MKN28, and SGC7901 and GES-1 cells was detected using western blot. All experiments were performed in triplicate. The results were expressed as mean ± SD. ^*∗*^*P* < 0.05, ^*∗∗*^*P* < 0.01, and ^*∗∗∗*^*P* < 0.001 versus normal group or GES-1.

Previous studies have demonstrated that POU2F1 plays a crucial role in promoting the progression of several tumors [10, 18, 29, 30]. To determine the expression of POU2F1 and TTC3-AS1 in GC, 10 paired gastric tumor tissues and adjacent normal tissues were detected using qRT-PCR and western blot. As shown in Figures 4(a)–4(c), the expression of both POU2F1 and TTC3-AS1 was significantly increased in the GC tissues (*P* < 0.05), compared with the adjacent normal tissues. Furthermore, the expression of POU2F1 and TTC3-AS1 were detected in five GC cell lines (MGC803, BGC823, MKN28, SGC7901, and normal GES-1). The results showed that both POU2F1 and TTC3-AS1 were significantly upregulated in the GC cell lines, compared with the normal GES-1 cells (Figures 4(d)–4(f)). Among these GC cell lines, SGC7901 exhibited the highest expression of POU2F1 and TTC3-AS1 and was used as a model for subsequent function assays. In summary, these results suggested that POU2F1 and TTC3-AS1 were highly expressed and predicted poor prognosis in GC.

### 3.2. lncRNA TTC3-AS1 Promoted the Cell Viability, Invasion, and Migration, but Had No Effect on POU2F1

**Figure 5:**
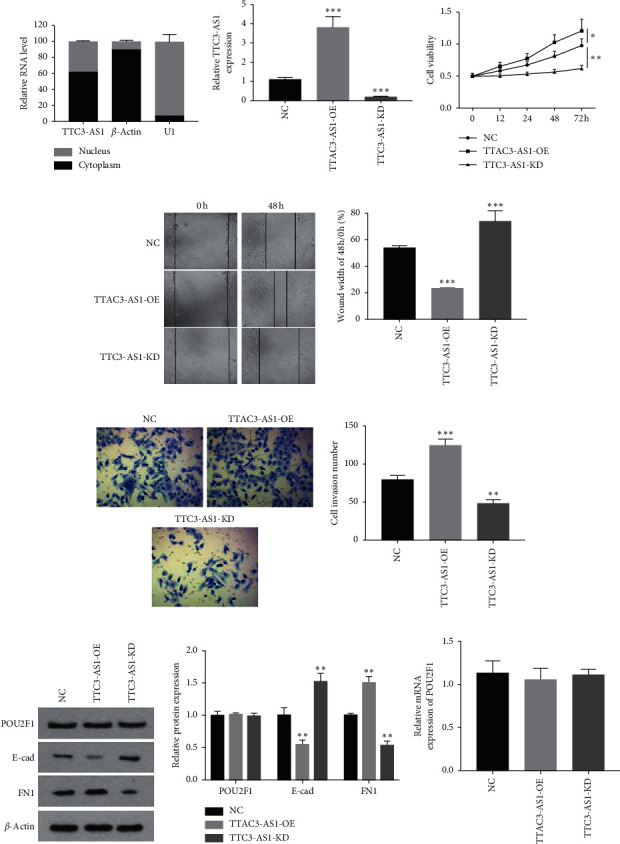
lncRNA TTC3-AS1 promoted the cell viability, invasion, and migration of GC cells but had no effect on POU2F1. (a) The subcellular distribution of lncRNA TTC3-AS1 in SGC7901 cells. (b) TTC3-AS1 was, respectively, knocked down by siRNA transfection and overexpressed by plasmid transfection; SGC7901 cells were divided into three groups, NC, TTC3-AS1-KD, and TTAC3-AS1-OE, and function experiments were performed. (c) Cell viability was detected using CCK-8 assays, and cell migration and invasion were, respectively, detected using (d) scratch assay and (e) transwell assay. (f) Protein expression of POU2F1, E-cad, and FN1 was detected using western blot. (g) The effects of TTC3-AS1 knockdown or overexpression on POU2F1 mRNA expression were evaluated using qRT-PCR. All experiments were performed in triplicate. The results were expressed as mean ± SD. ^*∗*^*P* < 0.05, ^*∗∗*^*P* < 0.01, and ^*∗∗∗*^*P* < 0.001 versus NC group.

To investigate the function of lncRNA TTC3-AS1 in GC progression, the subcellular distribution of lncRNA TTC3-AS1 in the cell was determined by PCR. The results in Figure 5(a) showed that lncRNA TTC3-AS1 was located in the cytoplasm mainly, but also in the cell nucleus, which suggested lncRNA TTC3-AS1 might be involved in posttranscriptional regulation of genes. Next, TTC3-AS1 siRNA (si-TTC3-AS1) and overexpression plasmids (TTC3-AS1-OE) were transfected into SGC7901 cells. The transfection efficiencies are shown in Figure 5(b), compared with the control group, TTC3-AS1-OE increased TTC3-AS1 expression to nearly 3.8-fold, and si-TTC3-AS1 decreased TTC3-AS1 expression to nearly 15%. Cell viability was detected using the CCK-8 assay (Figure 5(c)), which showed that TTC3-AS1-OE transfection significantly promoted the viability of SGC7901 cells (*P* < 0.05), while si-TTC3-AS1 exerted opposite effects (*P* < 0.01). Furthermore, transwell invasion and scratch assays also showed that cell invasion and migration were significantly increased in the TTC3-AS1-OE group while decreased in the si-TTC3-AS1 group, compared with the NC (nonspecific control) group (Figures 5(d) and 5(e); all *P* < 0.01). Moreover, the effects of TTC3-AS1 on the expression of POU2F1 and EMT- (epithelial-mesenchymal transition-) related proteins were investigated. As shown in Figure 5(f), TTC3-AS1-OE significantly decreased E-cad levels and increased FN1 levels compared with the NC (normal control) group, while si-TTC3-AS1 had opposite effects (all *P* < 0.05). However, lncRNA TTC3-AS1 had no effects on both mRNA and protein expression of POU2F1 (Figures 5(f) and 5(g)). To sum up, lncRNA TTC3-AS1 played an oncogenic role in GC, which was not mediated by POU2F1.

### 3.3. POU2F1 Promoted Cell Viability, Invasion, and Migration and Improved TTC3-AS1 Expression

**Figure 6:**
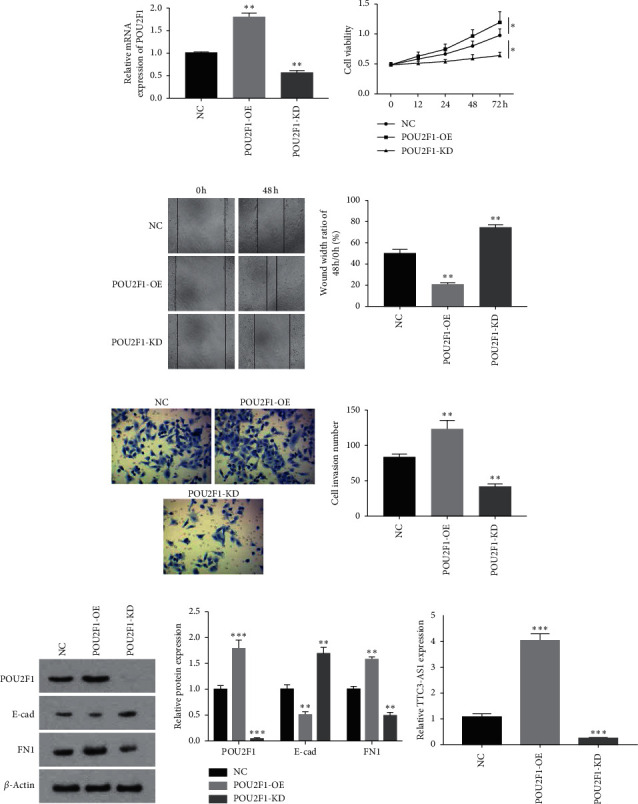
POU2F1 promoted GC cell viability, invasion, and migration of GC cells and promoted the expression of TTC3-AS1. (a) POU2F1 was, respectively, overexpressed by plasmid transfection and knocked down by siRNA transfection in SGC7901 cells; SGC7901 cells were divided into three groups, NC, POU2F1-KD, and POU2F1-OE, and function experiments were performed. (b) Cell viability was detected using CCK-8 assays, and cell migration and invasion were, respectively, detected using (c) scratch assay and (d) transwell assay. (e) Protein expression of POU2F1, E-cad, and FN1 was detected using western blot. (f) The effects of POU2F1 knockdown or overexpression on TTC3-AS1 expression were evaluated by using qRT-PCR. All experiments were performed in triplicate. The results were expressed as mean ± SD. ^*∗*^*P* < 0.05, ^*∗∗*^*P* < 0.01, and ^*∗∗∗*^*P* < 0.001 versus NC group.

The role of POU2F1 in GC progression was further investigated, and its possible interaction with TTC3-AS1 was determined. As shown in Figure 6(a), POU2F1-OE effectively increased the expression of POU2F1 and POU2F1-KD decreased its expression in SGC7901 cells. Function analysis indicated that POU2F1-OE significantly promoted the viability, invasion, and migration of SGC7901 cells, while POU2F1-KD inhibited these activities (Figures 6(b)–6(d)). Similarly, POU2F1 reduced E-cad expression and improved FN1 expression (Figure 6(e)). Then the expression of TTC3-AS1 was detected using qRT-PCR; the results revealed that it was significantly enhanced in the POU2F1-OE group and reduced in the POU2F1-KD group, compared with the NC group (both *P* < 0.05; Figure 6(f)). Collectively, POU2F1 functioned as an oncogene in GC, and the function was possibly mediated by lncRNA TTC3-AS1.

### 3.4. POU2F1 Directly Bound to the Promoter Region of TTC3-AS1

Based on the present evidence that transcription activator POU2F1 upregulated TTC3-AS1 expression, and CHIP and DAPA assays were performed to determine whether POU2F1 directly interacted with TTC3-AS1. Bioinformatics analysis revealed that there were two high-scoring POU2F1 binding sites on the upstream of the TTC3-AS1 promoter (Figure 1(a)). CHIP assays showed enrichment of the TTC3-AS1 promoter region in the anti-POU2F1 group, compared with the IgG control group (Figure 1(b)). Furthermore, overexpression of POU2F1 increased the enrichment of the TTC3-AS1 promoter region compared with the NC group (Figure 1(b)). DAPA assays, combined with western blot, suggested that the two wild-type (WT) sequences of TTC3-AS1 promoter could effectively capture POU2F1 proteins, while there was almost no POU2F1 enrichment in the mutant-type (MT) sequence group (Figure 1(c)). A dual-luciferase reporter assay was performed to confirm whether POU2F1 directly binds to the TTC3-AS1 promoter and to determine the binding sites of POU2F1 in the promoter region. Firstly, the introduction of POU2F1 significantly improved the luciferase activity compared with the TTC3-AS1-WT group (*P* < 0.01). When either of the two potential binding sites was mutated, the introduction of POU2F1 still improved the luciferase activity significantly compared with the TTC3-AS1-WT group (both *P* < 0.05), unless the two potential binding sites were mutated simultaneously (*P* > 0.05). Secondly, compared with the POU2F1 + TTC3-AS1-WT group, the luciferase activity in the POU2F1 + TTC3-AS1-MT1 (786–797 bp) group was significantly reduced (*P* < 0.05), while the luciferase activity in the POU2F1 + TTC3-AS1-MT2 (1066–1077 bp) group showed no notable variation (*P* > 0.05); mutation of the two potential binding sites also lessened the luciferase activity (*P* < 0.01), suggesting that POU2F1 prefers to target the site positioned at 786–797 bp in the TTC3-AS1 promoter region. In summary, POU2F1 could directly bind to the TTC3-AS1 promoter and promote its transcription activation.

### 3.5. lncRNA TTC3-AS1 Mediated the Oncogenic Function of POU2F1 in GC Cells

**Figure 7:**
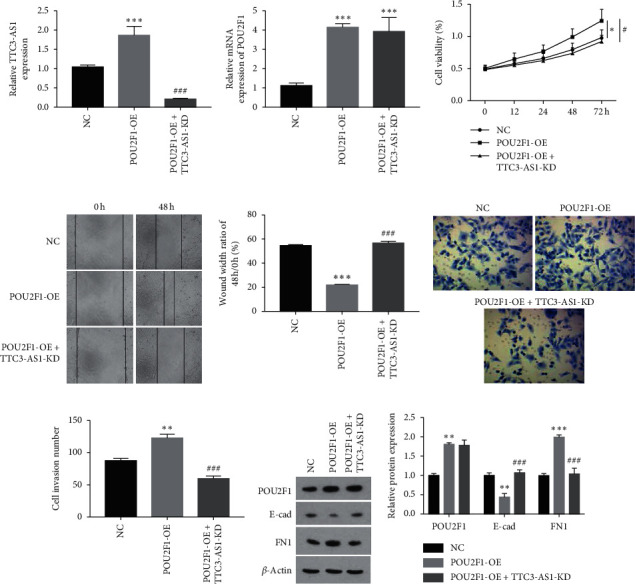
The oncogenic function of POU2F1 in GC cells was mediated by lncRNA TTC3-AS1. SGC7901 cells were divided into three groups, NC, POU2F1-OE, and POU2F1-OE + TTC3-AS1-KD, and rescue experiments were performed. The RNA expression of (a) lncRNA TTC3-AS1 and (b) POU2F1 was detected by using qRT-PCR. (c) Cell viability was detected by using CCK-8 assays, and cell migration and invasion were, respectively, detected by using (d) scratch assay and (e, f) transwell assay. (g) Protein expression of POU2F1, E-cad, and FN1 was detected by using western blot. (f) The effects of POU2F1 knockdown or overexpression on lncRNA TTC3-AS1 expression was evaluated by using qRT-PCR. All experiments were performed in triplicate. The results were expressed as mean ± SD. ^*∗*^*P* < 0.05, ^*∗∗*^*P* < 0.01, and ^*∗∗∗*^*P* < 0.001 versus NC group.^#^*P* < 0.05, ^##^*P* < 0.01, and ^###^*P* < 0.001 versus POU2F1-OE group.

Rescue experiments were performed in SGC7901 cells to confirm the lncRNA TTC3-AS1-mediated oncogenic function of POU2F1. SGC7901 cells were divided into three groups: (1) NC; (2) POU2F1-OE; and (3) POU2F1-OE + TTC3-AS1-KD. The results revealed that TTC3-AS1 expression was significantly enhanced by overexpression of POU2F1 but declined to a low level in POU2F1-OE + TTC3-AS1-KD group (Figure 7(a)). POU2F1 expression was dramatically elevated after overexpression of POU2F1 (*P* < 0.001), and cotransfection with TTC3-AS1-KD had no effect on the elevation (Figure 7(b)). Function analysis indicated that POU2F1 overexpression promoted the cell viability, migration, and invasion and activated EMT-related pathways, involving a decrease in E-cad levels and an increase in FN1 levels. In contrast, cotransfection of TTC3-AS1-KD and POU2F1-OE neutralized the protumor effects of POU2F1-OE (Figures 7(c)–7(g)). Summarily, lncRNA TTC3-AS1 mediated the oncogenic function of POU2F1 in GC cells.

### 3.6. POU2F1 Promoted GC Tumor Growth In Vivo through lncRNA TTC3-AS1

**Figure 8:**
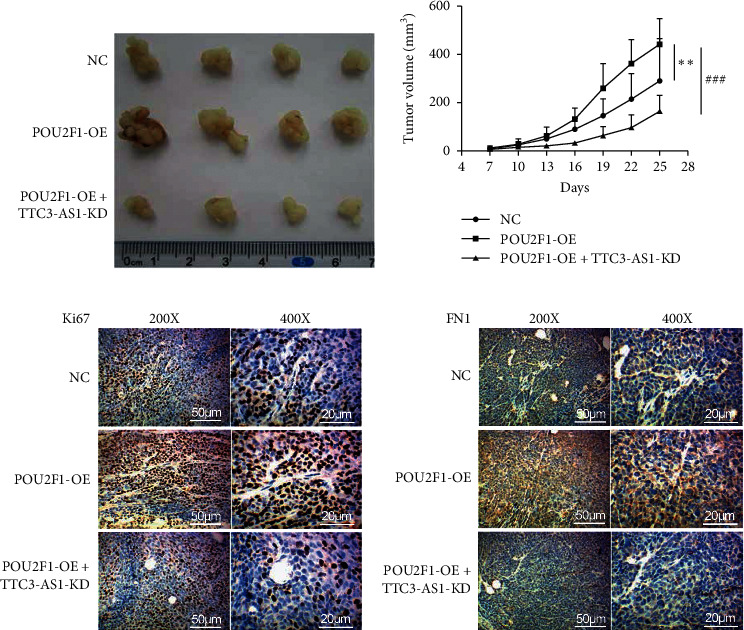
POU2F1 promoted GC tumor growth in vivo through TTC3-AS1. SGC7901 cells were, respectively, transfected by (1) NC, (2) POU2F1-OE, and (3) POU2F1-OE + TTC3-AS1-KD. Transfected SGC7901 cells were subcutaneously injected into the left flanks of male athymic BALB/c nude mice, and GC tumorigenesis was measured. (a) Tumor photographs in experiments end. (b) Tumor volume was detected. The expression of (c) Ki67 and (d) FN1 in GC tumor tissues was evaluated using IHC assays. All experiments were performed in triplicate. The results were expressed as mean ± SD. ^*∗*^*P* < 0.05, ^*∗∗*^*P* < 0.01, and ^*∗∗∗*^*P* < 0.001 versus NC group. ^#^*P* < 0.05, ^##^*P* < 0.01, and ^###^*P* < 0.001 versus POU2F1-OE group.

In order to investigate the tumor-growth function of POU2F1/TTC3-AS1 in GC, the transfected SGC7901 cells were subcutaneously injected into the left flanks of male athymic BALB/c nude mice, and GC tumorigenesis was evaluated. The mice were divided into three groups: (1) NC; (2) POU2F1-OE; and (3) POU2F1-OE + TTC3-AS1-KD. The results showed that GC tumor growth was significantly enhanced by POU2F1-OE, compared with the NC group, while cotransfection of TTC3-AS1-KD inhibited GC tumor growth (Figures 8(a) and 8(b)). IHC assays suggested that POU2F1-OE promoted the positive expression of Ki67 and FN1 compared with the NC group, while cotransfection of TTC3-AS1-KD reversed the effects (Figures 8(c) and 8(d)). In summary, POU2F1/TTC3-AS1 axis promoted tumor growth in GC.

## 4. Discussion

GC is one of the most frequently diagnosed cancers worldwide and causes numerous human deaths every year. In recent years, scientists have made great efforts to explore the mechanism involving tumorigenesis and development in GC, as well as abnormal gene expression in GC pathogenesis [31–33]. In the present study, through bioinformatic analysis of the HCMD-EXP00440 database, 5 lncRNAs (CATIP-AS2, TTC3-AS1, LINC01993, LINC01564, and LINC02015) and 11 specific TFs (POU5F1, CUX2, TBX19, POU2F1, PLAG1, ZFP57, LMX1A, MZF1, BACH1, ONECUT1, and IRF9) were identified to be significantly upregulated in metastatic tumor samples (*n* = 21), compared with primary tumor samples (*n* = 351). The results implied that these differentially expressed lncRNAs or mRNAs might play crucial roles in GC tumor metastasis. Then, whether the 11 TF genes had potential bind regions on the promoters of the 5 lncRNAs was determined using the JASPAR database. It was found that POU2F1 potentially targeted the promoter region of TTC3-AS1. POU2F1 is an important member of the POU (Pit-1, Oct1/2, and UNC-86) homeodomain family consisting of 13 POU TFs, and POU2F1 is the only POU family protein, which is widely expressed [14]. Analyses of public databases indicated that POU2F1 exhibited higher expression in renal, ovarian, and esophageal cancers while lower expression in the cerebral tumor, bladder cancer, and liposarcoma, compared with NCs. Recent studies have indicated that POU2F1 was highly expressed in GC patients [34–36]. In this study, we also identified that POU2F1 and its potential target TTC3-AS1 were significantly upregulated in GC tumor tissues, compared with normal tissues. Survival analysis revealed that high expression of POU5F1 was associated with shorter OS, DFS, and PFS, and TTC3-AS1 predicted poor DFS in GC patients. Our results indicated that POU5F1/TTC3-AS1 might play a crucial role in GC progression.

Increasing studies have revealed that POU2F1, as a multifunctional transcription factor, promoted tumorigenesis and progression through modulation of tumor-specific gene expression. For instance, in head and neck cancer, POU2F1 can bind to the promoters of HOXD10 and HOXD11 to activate their transcription, thus promoting tumor progression [17]. POU2F1 promotes growth and metastasis of hepatocellular carcinoma (HCC) through the FAT1 pathway [10]. With respect to GC, Xiao et al. [34] have reported that POU2F1 directly bound to the promoter of tumor suppressor miR-4490 and inhibited its transcription to promote GC development and metastasis. Moreover, phosphorylation-activated AKT upregulates the expression of POU2F1, thus improving the expression of ECD (ecdysoneless homologue) in GC [35]. It has been disclosed that POU2F1 promoted EMT by targeting TWIST1 and SLUG, which are part of a group of TFs inducing EMT in cancer cells, therefore facilitating the invasion and metastasis of cancer cells. Cell proliferation and migration as well as EMT were improved by POU2F1 overexpression in colorectal cancer (CRC), and the effects were reversed by POU2F1 knockdown. In addition, Spp1/Opn, as a POU2F1 target gene, encodes osteopontin reported to be highly expressed in several cancers involving GC. In breast cancer, osteopontin was found to promote tumor metastasis and development [14].

On the other hand, some studies found that POU2F1 is regulated by upstream signal molecules, especially the lncRNA/miRNA axis. For example, lncRNA CRNDE/miR-539-5p promotes HCC (hepatocellular carcinoma) progression through inhibition of POU2F1 [37]. Others such as ncRNA SND1-IT1/miRNA-665, TUG1/miR-9-5p, and NEAT1/miR-9-5p also play protumor roles through regulation of POU2F1 expression [16, 38, 39]. However, currently, there is no study exploring whether POU2F1 is involved in lncRNA expression in the tumor. Here, we found that POU2F1 was highly expressed in GC patients and promoted cell viability, invasion, migration in vitro, as well as tumor growth in vivo. Furthermore, we identified an lncRNA TTC3-AS1, which was transcriptionally activated by POU2F1. Function analysis revealed that lncRNA TTC3-AS1 mediated the protumor function of POU2F1 in GC.

lncRNAs have been demonstrated to play crucial roles in the occurrence and development of GC. For instance, lncRNA-RMRP exerts carcinogenesis by acting as a miR-206 sponge and serves as a novel biomarker in GC [40]. In another study, lncRNA ANCR was reported to promote invasion and migration of GC by regulating FoxO1 expression to inhibit macrophage M1 polarization [41]. However, there are still many functional lncRNAs unidentified in tumors. Here, we found an oncogenic lncRNA TTC3-AS1 in GC. So far, it has not been studied. Our results showed that TTC3-AS1 was upregulated in tumor specimens of GC patients, and promoted the viability, invasion, and migration of GC cells. Furthermore, we illustrated that POU2F1 could directly bind to the promoter region of TTC3-AS1 and activated its transcription. Rescue experiments revealed that TTC3-AS1 knockdown could reverse the protumor effects of POU2F1 in GC cells as well as tumor growth in vivo.

## 5. Conclusion

In conclusion, the study illustrated that POU2F1 promoted GC cell viability, invasion, and migration as well as tumor growth in vivo through direct transcription activation of TTC3-AS1. POU2F1/TTC3-AS1 was upregulated in GC tumor tissues and predicted poor prognosis in GC patients. This study provided two potential prognostic biomarkers and therapeutic targets in GC.

## Figures and Tables

**Table 1:** Primer sequences of qRT-PCR.

Gene name		Sequence (5′⟶ 3′)
POU2F1	Forward primer	ATGAACAATCCGTCAGAAACCAG
POU2F1	Reverse primer	GATGGAGATGTCCAAGGAAAGC
CUX2	Forward primer	CGAGACCTCCACACTTCGTG
CUX2	Reverse primer	TGTTTTTCCGCCTCATTTCTCTG
ONECUT1	Forward primer	AGCGTCGAACTCTACATGCAA
ONECUT1	Reverse primer	TGCTTTGGTACAAGTGCTTGAT
PLAG1	Forward primer	ATCACCTCCATACACACGACC
PLAG1	Reverse primer	AGCTTGGTATTGTAGTTCTTGCC
GAPDH	Forward primer	ACCCTGAAGTACCCCATCGAG
GAPDH	Reverse primer	AGCACAGCCTGGATAGCAAC
TTC3-AS1^a^	Forward primer	AAGGGCATCAAAAGCTTCGG
TTC3-AS1^a^	Reverse primer	GTCCTACACCAGCATCCGTA
POU2F1^a^	Forward primer	ATGAACAATCCGTCAGAAACCAG
POU2F1^a^	Reverse primer	GATGGAGATGTCCAAGGAAAGC
*β*-Actin^a^	Forward primer	ACCCTGAAGTACCCCATCGAG
*β*-Actin^a^	Reverse primer	AGCACAGCCTGGATAGCAAC
TTC3-AS1^b^	Forward primer	CCCAAGACTCACCCTCAACT
TTC3-AS1^b^	Reverse primer	AGGGGCTTGAGTGATTTCCA
GAPDH^b^	Forward primer	TACTAGCGGTTTTACGGGCG
GAPDH^b^	Reverse primer	TCGAACAGGAGGAGCAGAGAGCGA

qRT-PCR: quantitative reverse transcription-polymerase chain reaction. ^a^qRT-PCR was performed following the chromatin immunoprecipitation (ChIP) assay. ^b^Promoter primers.

## Data Availability

The datasets generated/analyzed in this study are available from the corresponding author upon reasonable request.

## References

[1] Siegel R., Naishadham D., Jemal A. (2013). Cancer statistics, 2013. *CA: A Cancer Journal for Clinicians*.

[2] Jing J.-J., Liu H.-Y., Hao J.-K. (2012). Gastric cancer incidence and mortality in Zhuanghe, China, between 2005 and 2010. *World Journal of Gastroenterology*.

[3] Nakajima T. (2002). Gastric cancer treatment guidelines in Japan. *Gastric Cancer*.

[4] Crew K. D., Neugut A. I. (2006). Epidemiology of gastric cancer. *World Journal of Gastroenterology*.

[5] Yang L. (2006). Incidence and mortality of gastric cancer in China. *World Journal of Gastroenterology*.

[6] Tantin D., Schild-Poulter C., Wang V., Haché R. J. G., Sharp P. A. (2005). The octamer binding transcription factor Oct-1 is a stress sensor. *Cancer Research*.

[7] Sive H. L., Roeder R. G. (1986). Interaction of a common factor with conserved promoter and enhancer sequences in histone H2B, immunoglobulin, and U2 small nuclear RNA (snRNA) genes. *Proceedings of the National Academy of Sciences*.

[8] Segil N., Roberts S., Heintz N. (1991). Mitotic phosphorylation of the Oct-1 homeodomain and regulation of Oct-1 DNA binding activity. *Science*.

[9] Fan W., Jin S., Tong T. (2002). BRCA1 regulates GADD45 through its interactions with the OCT-1 and CAAT motifs. *Journal of Biological Chemistry*.

[10] Zhu H. Y., Cao G. Y., Wang S. P. (2017). POU2F1 promotes growth and metastasis of hepatocellular carcinoma through the FAT1 signaling pathway. *American Journal of Cancer Research*.

[11] Li F., Wang T., Huang Y. (2021). POU2F1 induces the immune escape in lung cancer by up-regulating PD-L1. *American Journal of Translational Research*.

[12] Kim H., Dickey L., Stone C., Jafek J. L., Lane T. E., Tantin D. (2019). T cell-selective deletion of Oct1 protects animals from autoimmune neuroinflammation while maintaining neurotropic pathogen response. *Journal of Neuroinflammation*.

[13] Fontela M. G., Notario L., Alari-Pahissa E., Lorente E., Lauzurica P. (2019). The conserved non-coding sequence 2 (CNS2) enhances CD69 transcription through cooperation between the transcription factors Oct1 and RUNX1. *Genes*.

[14] Vázquez-Arreguín K., Tantin D. (2016). The Oct1 transcription factor and epithelial malignancies: old protein learns new tricks. *Biochimica et Biophysica Acta (BBA)—Gene Regulatory Mechanisms*.

[15] Jeong S.-H., Lee Y.-J., Cho B.-I. (2014). OCT-1 overexpression is associated with poor prognosis in patients with well-differentiated gastric cancer. *Tumor Biology*.

[16] Xie C.-H., Cao Y.-M., Huang Y. (2016). Long non-coding RNA TUG1 contributes to tumorigenesis of human osteosarcoma by sponging miR-9-5p and regulating POU2F1 expression. *Tumor Biology*.

[17] Sharpe D. J., Orr K. S., Moran M. (2014). POU2F1 activity regulates HOXD10 and HOXD11 promoting a proliferative and invasive phenotype in head and neck cancer. *Oncotarget*.

[18] Xiao Y., Liu S., Li J. (2020). The POU2F1/miR-4490/USP22 axis regulates cell proliferation and metastasis in gastric cancer. *Cell Oncology*.

[19] Abbastabar M., Sarfi M., Golestani A., Khalili E. (2018). lncRNA involvement in hepatocellular carcinoma metastasis and prognosis. *EXCLI Journal*.

[20] Zhou N., He Z., Tang H., Jiang B., Cheng W. (2019). LncRNA RMRP/miR-613 axis is associated with poor prognosis and enhances the tumorigenesis of hepatocellular carcinoma by impacting oncogenic phenotypes. *American Journal of Translational Research*.

[21] Hong L., Wang H., Wang J. (2019). LncRNA PTCSC3 inhibits tumor growth and cancer cell stemness in gastric cancer by interacting with lncRNA linc-pint. *Cancer Management and Research*.

[22] Li D., Wang J., Zhang M. (2020). LncRNA MAGI2-AS3 is regulated by BRD4 and promotes gastric cancer progression via maintaining ZEB1 overexpression by sponging miR-141/200a. *Molecular Therapy—Nucleic Acids*.

[23] Zhu L., Jia R., Zhang J., Li X., Qin C., Zhao Q. (2020). Quantitative proteomics analysis revealed the potential role of lncRNA ftx in promoting gastric cancer progression. *Proteomics. Clinical Applications*.

[24] Shi X. Y., Sun Y. Z., Li M., Li H. Y. (2019). LncRNA CADM1-AS1 serves as a new prognostic biomarker for gastric cancer. *European Review for Medical and Pharmacological Sciences*.

[25] Qu C. X., Shi X. C., Bi H., Zhai L. Q., Yang Q. (2019). LncRNA AOC4P affects biological behavior of gastric cancer cells through MAPK signaling pathway. *European Review for Medical and Pharmacological Sciences*.

[26] Schmittgen T. D., Livak K. J. (2008). Analyzing real-time PCR data by the comparative CT method. *Nature Protocols*.

[27] Billon N., Carlisi D., Datto M. B. (1999). Cooperation of Sp1 and p300 in the induction of the CDK inhibitor p21WAF1/CIP1 during NGF-mediated neuronal differentiation. *Oncogene*.

[28] Chen D. L., Zeng Z. L., Yang J. (2013). L1cam promotes tumor progression and metastasis and is an independent unfavorable prognostic factor in gastric cancer. *Journal of Hematology & Oncology*.

[29] Zhong Y., Huang H., Chen M. (2017). POU2F1 over-expression correlates with poor prognoses and promotes cell growth and epithelial-to-mesenchymal transition in hepatocellular carcinoma. *Oncotarget*.

[30] Zhang R., Lu H., Lyu Y. Y. (2017). E6/E7-P53-POU2F1-CTHRC1 axis promotes cervical cancer metastasis and activates Wnt/PCP pathway. *Scientific Reports*.

[31] Camargo M. C., Figueiredo C., Machado J. C. (2019). Review: gastric malignancies: basic aspects. *Helicobacter*.

[32] Smyth E. C., Nilsson M., Grabsch H. I., van Grieken N. C., Lordick F. (2020). Gastric cancer. *The Lancet*.

[33] Strong V. E. (2018). Progress in gastric cancer. *Updates in Surgery*.

[34] Xiao Y., Liu S., Li J. (2020). The POU2F1/miR-4490/USP22 axis regulates cell proliferation and metastasis in gastric cancer. *Cellular Oncology*.

[35] Xu S.-H., Huang J.-Z., Xu M.-L. (2015). ACK1 promotes gastric cancer epithelial-mesenchymal transition and metastasis through AKT-POU2F1-ECD signalling. *The Journal of Pathology*.

[36] Liang Y., Zhang C.-D., Zhang C., Dai D.-Q. (2020). DLX6-AS1/miR-204-5p/OCT1 positive feedback loop promotes tumor progression and epithelial-mesenchymal transition in gastric cancer. *Gastric Cancer*.

[37] Li Z., Wu G., Li J., Wang Y., Ju X., Jiang W. (2020). lncRNA CRNDE promotes the proliferation and metastasis by acting as sponge miR-539-5p to regulate POU2F1 expression in HCC. *BMC Cancer*.

[38] Xie Q., Lin S., Zheng M., Cai Q., Tu Y. (2019). Long noncoding RNA NEAT1 promotes the growth of cervical cancer cells via sponging miR-9-5p. *Biochemistry and Cell Biology*.

[39] Jin X. M., Xu B., Zhang Y. (2019). LncRNA SND1-IT1 accelerates the proliferation and migration of osteosarcoma via sponging miRNA-665 to upregulate POU2F1. *European Review for Medical and Pharmacological Sciences*.

[40] Shao Y., Ye M., Li Q. (2016). LncRNA-RMRP promotes carcinogenesis by acting as a miR-206 sponge and is used as a novel biomarker for gastric cancer. *Oncotarget*.

[41] Xie C., Guo Y., Lou S. (2020). LncRNA ANCR promotes invasion and migration of gastric cancer by regulating FoxO1 expression to inhibit macrophage M1 polarization. *Digestive Diseases and Sciences*.

